# Telomere-specific chromatin capture using a pyrrole–imidazole polyamide probe for the identification of proteins and non-coding RNAs

**DOI:** 10.1186/s13072-021-00421-8

**Published:** 2021-10-09

**Authors:** Satoru Ide, Asuka Sasaki, Yusuke Kawamoto, Toshikazu Bando, Hiroshi Sugiyama, Kazuhiro Maeshima

**Affiliations:** 1 Genome Dynamics Laboratory, National Institute of Genetics, ROIS, Mishima, Shizuoka 411-8540 Japan; 2 Department of Genetics, School of Life Science, SOKENDAI, Mishima, Shizuoka 411-8540 Japan; 3 Department of Chemistry, Graduate School of Science, Kyoto University, Sakyo, Kyoto, 606-8502 Japan

**Keywords:** Chromatin, Chromatin purification, Pyrrole–imidazole (PI) polyamide, Non-coding RNA, Telomere, ALT (alternative lengthening of telomeres)

## Abstract

**Background:**

Knowing chromatin components at a DNA regulatory element at any given time is essential for understanding how the element works during cellular proliferation, differentiation and development. A region-specific chromatin purification is an invaluable approach to dissecting the comprehensive chromatin composition at a particular region. Several methods (e.g., PICh, enChIP, CAPTURE and CLASP) have been developed for isolating and analyzing chromatin components. However, all of them have some shortcomings in identifying non-coding RNA associated with DNA regulatory elements.

**Results:**

We have developed a new approach for affinity purification of specific chromatin segments employing an *N*-methyl pyrrole (P)-*N*-methylimidazole (I) (PI) polyamide probe, which binds to a specific sequence in double-stranded DNA via Watson–Crick base pairing as a minor groove binder. This new technique is called proteomics and RNA-omics of isolated chromatin segments (PI-PRICh). Using PI-PRICh to isolate mouse and human telomeric components, we found enrichments of shelterin proteins, the well-known telomerase RNA component (TERC) and telomeric repeat-containing RNA (TERRA). When PI-PRICh was performed for alternative lengthening of telomere (ALT) cells with highly recombinogenic telomeres, in addition to the conventional telomeric chromatin, we obtained chromatin regions containing telomeric repeat insertions scattered in the genome and their associated RNAs.

**Conclusion:**

PI-PRICh reproducibly identified both the protein and RNA components of telomeric chromatin when targeting telomere repeats. PI polyamide is a promising alternative to simultaneously isolate associated proteins and RNAs of sequence-specific chromatin regions under native conditions, allowing better understanding of chromatin organization and functions within the cell.

**Supplementary Information:**

The online version contains supplementary material available at 10.1186/s13072-021-00421-8.

## Background

Genome activities, such as transcription and replication, are often achieved by DNA regulatory elements interacting with protein complexes and modified histone proteins to shape numerous and unique chromatin landscapes [1, 2]. Recently, non-coding RNAs (ncRNAs), which are defined as RNAs that are not translated into functional proteins, have emerged as key regulators of chromatin states and play roles in diverse biological processes, including X-chromosome dosage compensation, developmental gene expression, and chromosome stability through telomere elongation [3–8]. While chromatin immunoprecipitation (ChIP) is used to map protein–DNA interactions, genome-wide analyses of ncRNA-binding sites, such as CHART, ChIRP and RAP, have been developed to map chromatin-associated RNAs [9–11]. These latter assays utilize antisense oligonucleotide probes to retrieve specific ncRNAs with bound DNA sequences. However, these approaches require prior knowledge of the target ncRNA to assay. Methods for comprehensive and unbiased identification of ncRNAs associated with specific DNA regulatory elements are still in need.

Knowing all the players acting at DNA regulatory elements is a major step toward understanding how the element works. Several approaches are available for isolating and analyzing chromatin at specific regions [12]. We focus on chromatin isolation techniques targeting telomeres consisting of a long array of repetitive sequences (TTAGGG) in mammals, because the chromatin composition of the telomere is well known. One technique is a region-specific chromatin purification called PICh [13, 14]. In PICh, a locked nucleic acid (LNA) probe specifically targeted telomeric chromatin via Watson–Crick base pairing and was used to identify telomere-bound proteins in combination with mass spectrometry [13]. The quantitative telomeric chromatin isolation protocol (QTIP) is an antibody-based method employed to detect proteins bound to telomeres, as it can compare telomere-bound proteins isolated from cells in a variety of states using isotope labeling by amino acids in cell culture (SILAC) [15]. Both techniques efficiently enriched telomeric chromatin but were limited to only analyzing the protein components of the telomere. Apart from the oligonucleotide and antibody probes, the recent development of engineered DNA-binding molecules provides alternative methods for purifying telomeric chromatin. A transcription activator-like effector (TALE)-based strategy or the CRISPR system, which contains nuclease-deficient Cas9 protein and sequence-specific guide RNA, have been used in combination with enChIP [16], CAPTURE [17] and CLASP [18] to identify both telomere-specific protein complexes and also ncRNAs. However, these techniques had less than ten-fold enrichment of telomeric proteins, such as the shelterin complex. Accordingly, the methods still seem primitive for accurate quantification and de novo discovery of chromatin-associated RNAs.

We have previously developed *N*-methyl pyrrole (P)-*N*-methylimidazole (I) (PI) polyamides for the visualization of telomeres and the assessment of telomere length [19–26]. PI polyamide binds to the minor groove of double-stranded DNA without denaturation and can recognize Watson–Crick base pairs [27–34]. We have demonstrated that fluorescently labeled tandem hairpin PI polyamide (TH-59) that targets human telomere sequences (TTAGGG)_n_ can stain telomeres in cultured cells and tissue sections [19, 20, 35]. PI polyamide may be a more advantageous alternative to the nucleic acid probes used in any genomic analyses, including region-specific chromatin purification, because PI polyamide can target and bind a specific DNA sequence under mild conditions without non-specific binding to single-stranded RNA.

**Fig. 1:**
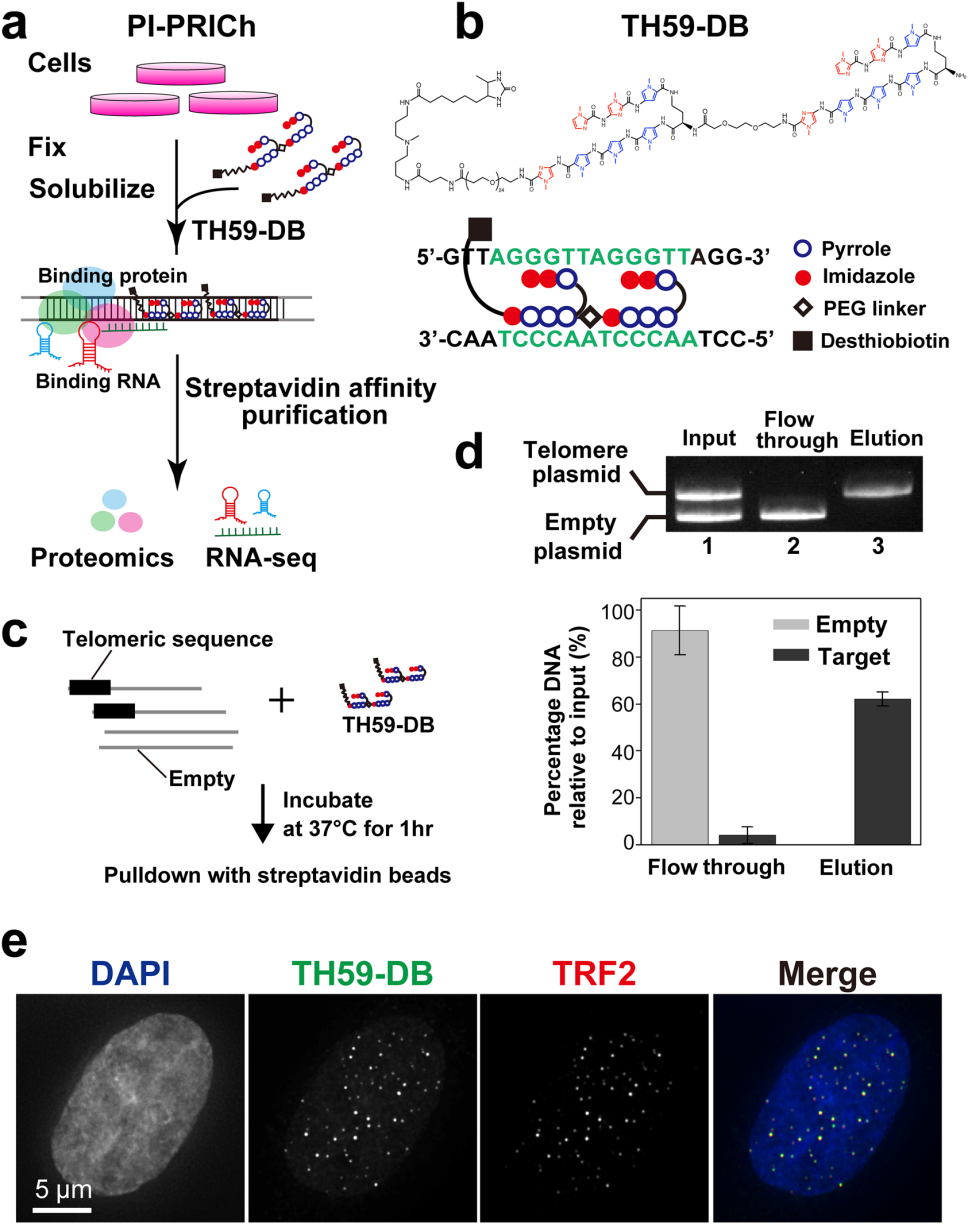
Affinity purification procedure of telomeric chromatin by a PI polyamide probe (PI-PRICh). **a** Scheme for telomeric chromatin isolation using a telomere-targeting PI polyamide probe (TH59-DB). The crude chromatin fraction is mixed and incubated with TH59-DB, and the probe-chromatin complexes are isolated by streptavidin affinity purification. The isolated chromatin fractions are analyzed by mass spectrometry for the study of proteins and by next-generation sequencing for the study of ncRNAs. **b** Chemical structure of TH59-DB. The base recognition profile of TH59-DB is shown in the lower part. **c** Outline of the plasmid pull-down assay. Linearized plasmids (gray line) with or without the telomeric repeat (thick black line) were mixed with TH59-DB. The mixture was incubated at 37 °C for binding of TH59-DB. Plasmid-TH59-DB hybrids were captured using MyOne C1 streptavidin beads. **d** Purification of the telomeric repeat-containing plasmid with TH59-DB. (top) Each fraction (Input, Flow-through, and Elution) was analyzed by agarose gel electrophoresis and EtBr staining. The positions of the telomeric repeat-containing plasmid and the empty vector are indicated. (bottom) Bar graph quantifying telomeric DNA capture. Error bars represent standard deviations. **e** Telomere labeling with TH59-DB in HeLa1.3 cells. Cells were stained with DAPI (first column), TH59-DB (second column) and anti-TRF2 antibody (third column). The merged images are in the fourth column

Here, we show a new region-specific chromatin purification method, using a PI polyamide probe, named Proteomics and RNA-omics of Isolated Chromatin segments (PI-PRICh) (Fig. 1a). We identified telomere-bound proteins from mouse erythrocyte leukemia (MEL) cells, such as the shelterin complex (TRF1, TRF2, POT1a, and TIN2), using PI-PRICh in combination with mass spectrometric analysis. At the same time, we also extracted the RNA fraction associated with telomeric chromatin and used next-generation sequencing (NGS) to comprehensively identify telomeric chromatin-associated RNAs. PI-PRICh mainly identified three types of ncRNAs involved in telomere maintenance: the telomerase RNA component (TERC) [36, 37], the telomeric repeat-containing RNA called TERRA (reviewed in [38, 39]) and ncRNAs transcribed from subtelomeric regions. Lastly, we discovered ncRNAs associated with the de novo telomeric sequence inserted into several intron regions when using PI-PRICh in alternative lengthening of telomere (ALT) cells, whose telomeres are highly recombinogenic. PI polyamides are thus a promising alternative for identifying proteins and ncRNAs to characterize chromatin at specific regions.

## Results

### PI polyamide probe for affinity purification of telomeric repeats DNA

We developed a method, PI-PRICh, which allows unbiased high-throughput identifications of telomeric chromatin-bound proteins and RNAs (Fig. 1a). For this purpose, we used a telomere-targeting tandem hairpin PI polyamide (TH59-DB), which has a biotin analog for affinity purification (Fig. 1b, Additional file 1: Fig. S1a and b). We first confirmed TH59-DB efficiently pulled down a plasmid containing a 750-bp telomeric repeats fragment, whereas the empty plasmid was not retrieved (Fig. 1c, d). Moreover, using streptavidin covalently attached to a fluorescent dye, TH59-DB targeted the telomere regions in the cells crosslinked with formaldehyde, which were co-immunostained by the TRF2 antibody (Fig. 1e). After verifying the targeting and binding specificity of TH59-DB, we isolated telomeric chromatin from crosslinked cells. Briefly, cultured cells were crosslinked, and their chromatin was extracted and homogenized. TH59-DB was bound to chromatin containing telomeric repeats. TH59-DB-bound chromatin was isolated using magnetic streptavidin beads. The co-purified proteins and RNAs were eluted and then subjected to downstream assays for identification and quantitation (Fig. 1a).

### Affinity purification of telomeric chromatin by the PI polyamide probe

**Fig. 2:**
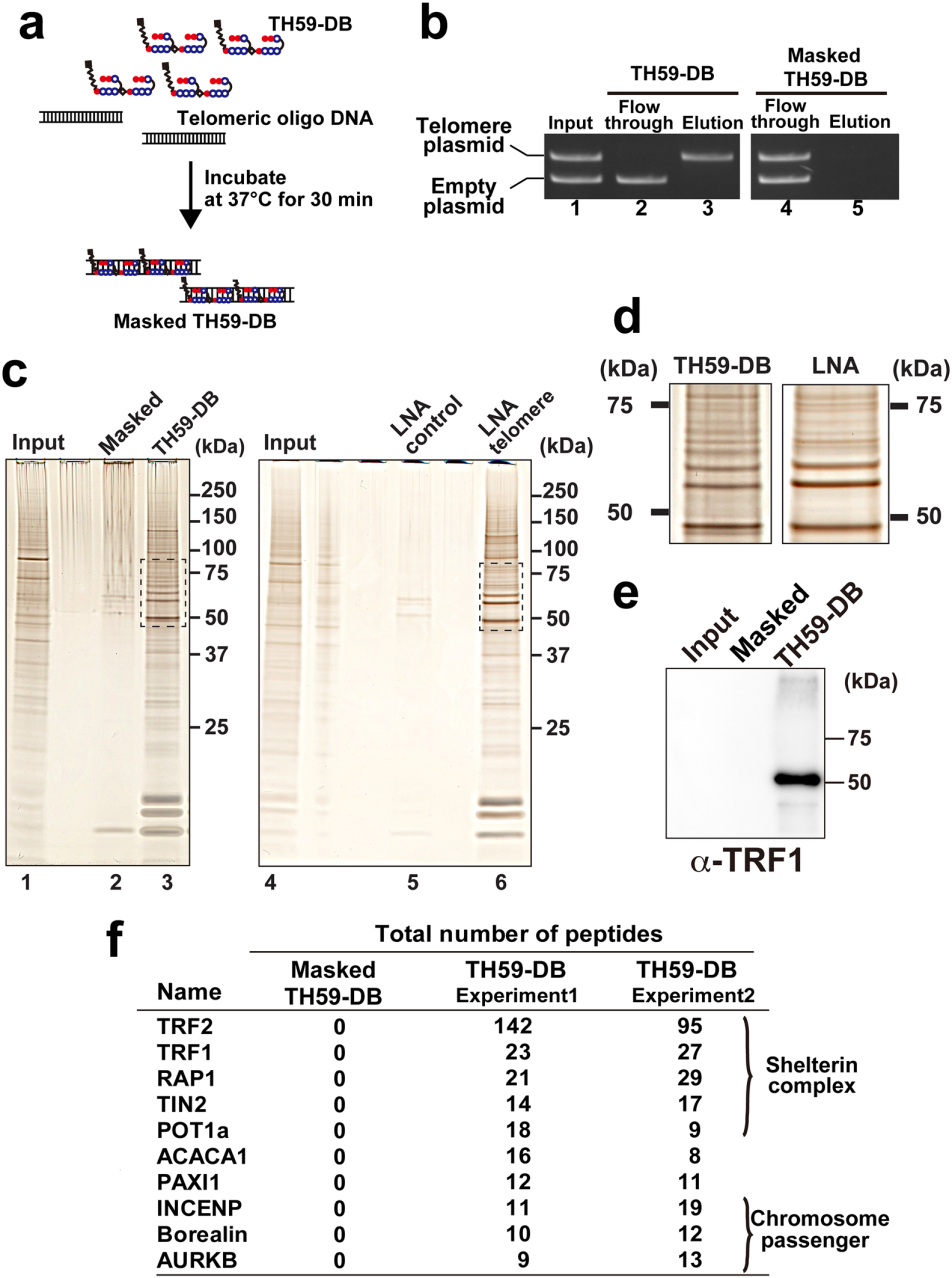
Affinity purification of the telomeric chromatin from mouse erythrocyte leukemia (MEL) cells by PI-PRICh. **a** Preparation of a negative control probe, masked TH59-DB, for telomeric chromatin isolation. TH59-DB was mixed and incubated with 50 times excess double-stranded oligonucleotide (TTAGGG)_4_/(CCCTAA)_4_ (telomeric oligonucleotides). **b** Purification validation of the telomeric repeat DNA with TH59-DB and masked TH59-DB. Telomeric repeat-containing plasmids were purified with TH59-DB (lane 2, 3) but not masked TH59-DB (lane 4, 5). Each fraction (Input, Flow-through, Elution) was analyzed by agarose gel electrophoresis and EtBr staining. The positions of the telomeric repeat-containing plasmid and the empty vector are indicated. **c** Silver staining of proteins obtained from telomeric chromatin purification with TH59-DB or LNA probes for the telomere repeat. (left) Chromatin isolation was performed with masked TH59-DB and TH59-DB. Input representing 0.001% of the starting material (10^4^ cells equivalent, lane 1), 8% of the masked TH59-DB pull-down fraction (lane 2) and of the TH59 pull-down fraction (lane 3). (right) Silver staining of proteins obtained from PICh with scrambled (LNA control, lane 5) or telomere LNA probes (LNA telomere, lane 6). **d** Enlarged images of the boxed region between 50 and 75 kDa in **c** to show their similar band patterns. **e** Western blot analysis for TRF1 in each fraction of PI-PRICh. Input representing 0.0005% of the chromatin extracts, 4% of the materials of masked TH59-DB pull-down fraction or TH59-DB pull-down fraction. **f** List of proteins detected by mass spectrometry analysis of the material purified by TH59-DB from MEL cells. The results from two independent experiments are shown. The top ten proteins are sorted by the total number of peptides

We isolated telomeric chromatin from MEL cells using TH59-DB. As a negative control probe, we premixed TH59-DB with telomeric oligo DNA prior to incubation with the chromatin extract (masked TH59-DB, Fig. 2a). The masked TH59-DB failed to capture telomeric DNA in the plasmid pull-down assay (Fig. 2b). TH59-DB and masked TH59-DB were incubated with solubilized MEL-derived chromatin and subsequently washed as described in Methods. Pull-down fractions were electrophoresed and analyzed by silver staining for protein analysis. Several specific bands were detected in the TH59-DB pull-down fraction, but not in the masked TH59-DB pull-down fractions (Fig. 2c, d). The banding pattern resembled that of telomeric proteins purified by PICh, based on LNA (Fig. 2c, d). To verify the specific enrichment of telomeric proteins, we monitored the presence of the known telomere-associating protein TRF1 by immunoblotting. TRF1 was highly enriched in the TH59-DB pull-down fraction but not in the masked TH59-DB pull-down (negative control) or input fractions (Fig. 2e). Importantly, we identified known telomeric proteins using mass spectrometry, such as shelterin components and chromosome passenger complexes (two independent results in Fig. 2f and Additional file 7: Table S1). Out of 32 proteins reproducibly identified by PI-PRICh, 30 proteins were found in the published PICh telomere data for mouse embryonic stem cells [40] (Additional file 7: Table S1). These data demonstrated that TH59-DB specifically bound to the telomeric sequence and efficiently purified telomeric chromatin.

### Comprehensive identification of telomeric chromatin-associated RNAs

**Fig. 3:**
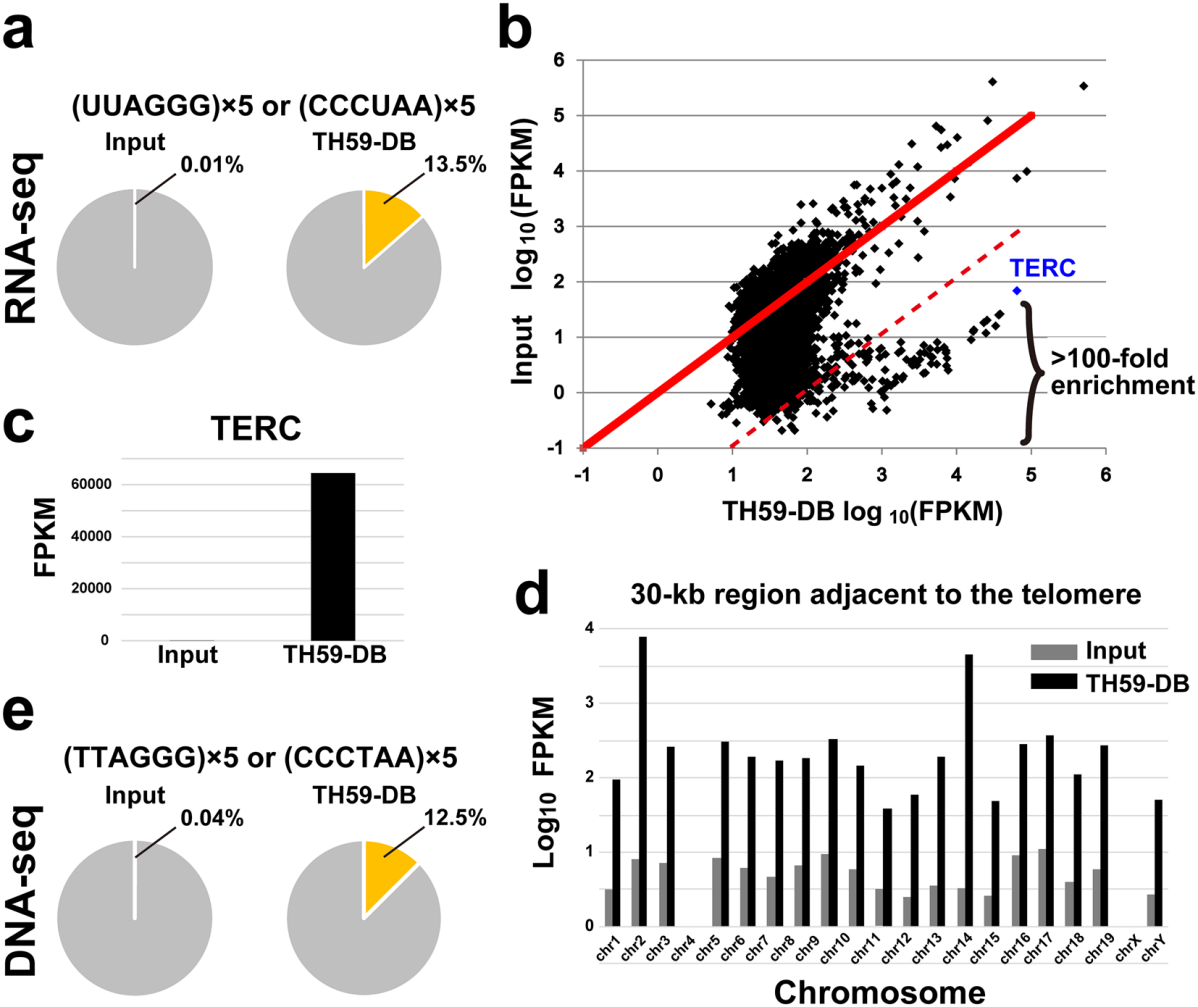
Comprehensive identification of telomeric chromatin-associated RNAs in MEL cells. **a** The percentage (yellow) of telomeric repeat reads, including TERRA transcripts, in the input and TH59-DB pull-down fractions from MEL cells. The number of the single-end reads, including (TTAGGG)_5_ or (CCCTAA)_5_, extracted from input and TH59-DB pull-down fractions were divided by the total number of reads. The gray color shows the other reads. **b** Scatter plot of fragments per kilobase of per million mapped reads (FPKM) of the TH59-DB pull-down fraction versus that of the input sample for each RNA. The telomerase RNA component, TERC (most enriched in the TH59-DB pull-down fraction), is highlighted in blue. RNAs that enriched more than 100-fold in TH59-DB pull-down fraction are plotted below a red dotted line. **c** Bar graph of FPKM of telomerase RNA-component (TERC) in input and TH59-DB pull-down fractions. **d** Bar graph of FPKM value of the transcripts at a 30-kb region adjacent to the telomere on the q arm of each chromosome in input and TH59-DB pull-down fractions. **e** Sequence analysis of DNA fragments captured by TH59-DB. The percentage (yellow) of telomeric repeat reads including (TTAGGG)_5_ or (CCCTAA)_5_, in the input and TH59-DB pull-down fractions from MEL cells. Note that the results of the second experiment (Experiment 2) are shown in Additional file 2: Figure S2

RNA was extracted from the TH59-DB pull-down fraction containing telomeric chromatin and subjected to NGS analysis, herein called RNA-Seq. Sequencing and mapping of these libraries yielded approximately 5.5 million (total) and 2 million (mappable) read pairs in the TH59-DB pull-down fraction and 7.8 million (total) and 3.5 million (mappable) read pairs in input. In the TH59-DB pull-down fraction, the substantial part of RNAs in our dataset (742249 out of 5479729, 13.5%) had > 5 times-repeated telomeric sequences, which corresponded to the telomeric repeat-containing RNAs such as TERRA and ARRET (Fig. 3a). The reads containing (TTAGGG)_5_ in the TH59-DB pull-down fraction were enriched 1000-fold over that of the input fraction (0.01%), suggesting PI-PRICh has a much higher purity of telomeric materials than that previously reported [16]. About 66% of the telomeric repeat-containing RNAs are TERRA and the remaining are ARRET, which is consistent with the previous reports that TERRA is more abundant than ARRET [8, 41].

To identify RNA highly enriched in the telomeric chromatin, we plotted RNA levels in the pull-down fraction (after purification) against that of input (before purification). As shown in Fig. 3b, a cluster of RNAs was enriched 100-fold over that of input. The most enriched RNA was the telomerase RNA component TERC (blue diamond in Fig. 3b, c).

Notably, almost all other RNAs also included (TTAGGG)_n_ repeat sequences (Additional file 8: Table S2). Such highly enriched RNAs might be derived from subtelomere regions, because telomeric repeats often exist in these regions [42]. Indeed, some sequence reads in the TH59-DB pull-down fraction were specifically mapped within the last 30-kb region adjacent to the telomere sequence of each chromosome (Fig. 3d and Additional file 1: Fig. S1c), which are considered to be subtelomeric regions on the q arm of each chromosome [43]. A part of the sequence reads was mapped to a validated TERRA transcript from the subtelomeric regions [44] (Fig. 3d and Additional file 1: Fig. S1c).

To assess the reproducibility of the method, we repeated the telomeric chromatin purification from a different batch of MEL cells using TH59-DB, and obtained similar TH59-DB pull-down fraction results (> 10% (39.1%) of RNAs had > 5 times-repeated telomeric sequences, and TERC was enriched 100-fold over that of input) (Additional file 2: Fig. S2a, b, c). Moreover, we analyzed DNA fragments by DNA-Seq from two independent TH59-DB pull-down fractions and estimated the enrichment of the target chromatin. 12.5% (Experiment 1) and 16.3% (Experiment 2) of the reads in each of the TH59-DB pull-down fractions had > 5 times-repeated telomeric sequences, indicating about a 300–350-fold enrichment of telomeric TTAGGG repeats (Fig. 3e and Additional file 2: Fig. S2d).

Altogether, we concluded that the PI polyamide probe based telomeric chromatin purification was superior to RNA-omics for telomeric chromatin-associated ncRNA, which has never been achieved by PICh using LNA probes.

### ncRNAs associated with telomeric repeats in ALT

We then focused on ALT cells, which are telomerase-negative and have highly recombinogenic telomeres. ALT telomeres have a different chromatin state from telomerase-driven telomeres [45]. Telomeric RNA, particularly TERRA, is highly expressed and might be a key player to promote homologous recombination in ALT cells [46, 47]. Additionally, telomere elongation by ALT is associated with genomic alternation via the telomeric repeats insertion into non-telomeric genome regions [48–50]. Based on these findings, we expected to pull-down chromatin regions containing the telomeric repeat insertions distributed in the genome as well as in telomeric chromatin.

**Fig. 4:**
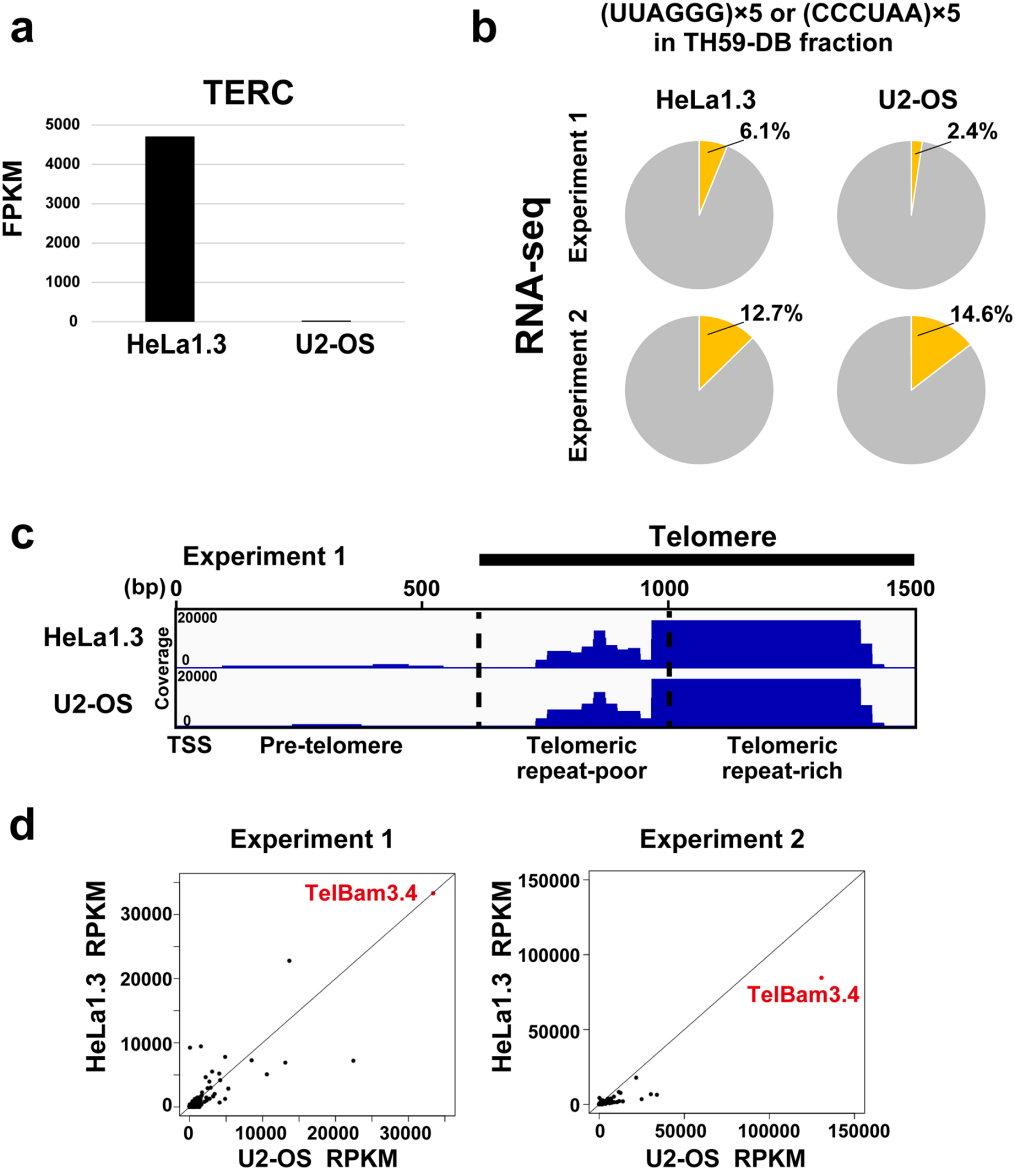
Comparison of the telomeric chromatin-associated TERRA between HeLa1.3 and ALT U2-OS cell lines. **a** Bar graph of FPKM of the telomerase RNA component (TERC) in telomerase-maintained telomeres (HeLa1.3) and ALT telomeres (U2-OS). **b** Pie charts show the percentage of telomere repeat sequence reads including TERRA/ARRET in the input and TH59-DB pull-down fractions from HeLa1.3 and U2-OS. Upper part and lower part show experiment 1 and experiment 2, respectively. **c** A browser view of chromatin-associated TERRA transcripts at a subtelomeric region downstream transcription start site (TSS) coding TelBam3.4 sequence using Integrative Genomic Viewer (IGV) in HeLa1.3 or U2-OS. The numbers on the left side in tracks indicate the read counts in RNA-seq. Telomere region in TelBam3.4 that was defined previously (Nergadze 2009, Usui 2015). Borderlines (vertical dotted black lines) are shown between pre-telomere, telomeric repeat-poor, and -rich regions based on TTAGGG repeat density. **d** Scatter plots of read per kilobase with normalized count per million (RPKM) in U2-OS cells versus that of HeLa1.3 cells for each RNA from two independent PI-PRICh experiments. RNA that is transcribed from the subtelomeric region of TelBam3.4 is highlighted in red

We applied PI-PRICh to U2-OS cells (ALT telomeres) and to HeLa1.3 cells (long telomeres with active telomerase). PI-PRICh data detected TERC in HeLa1.3 cells, but very little was found in U2-OS cells (Fig. 4a). We compared the amounts of TERRA in the pull-down fractions between the two cell lines and found little difference in their percentages of the reads, including > (TTAGGG)_5_ (Fig. 4b). Furthermore, the TERRA amount calculated from the mapped reads in U2-OS cells was comparable to that of HeLa1.3 cells (Fig. 4c, d) when we mapped the reads on a reference sequence of the subtelomeric region called TelBam3.4 [51, 52]. Others have suggested that TERRA can trigger recombination through the hybridization with telomeric DNA as TERRA appears to be expressed in ALT cells more than telomerase-positive cells [46, 47]. However, our results imply that not all TERRA RNAs bind to telomere repeats in ALT cells.

**Fig. 5:**
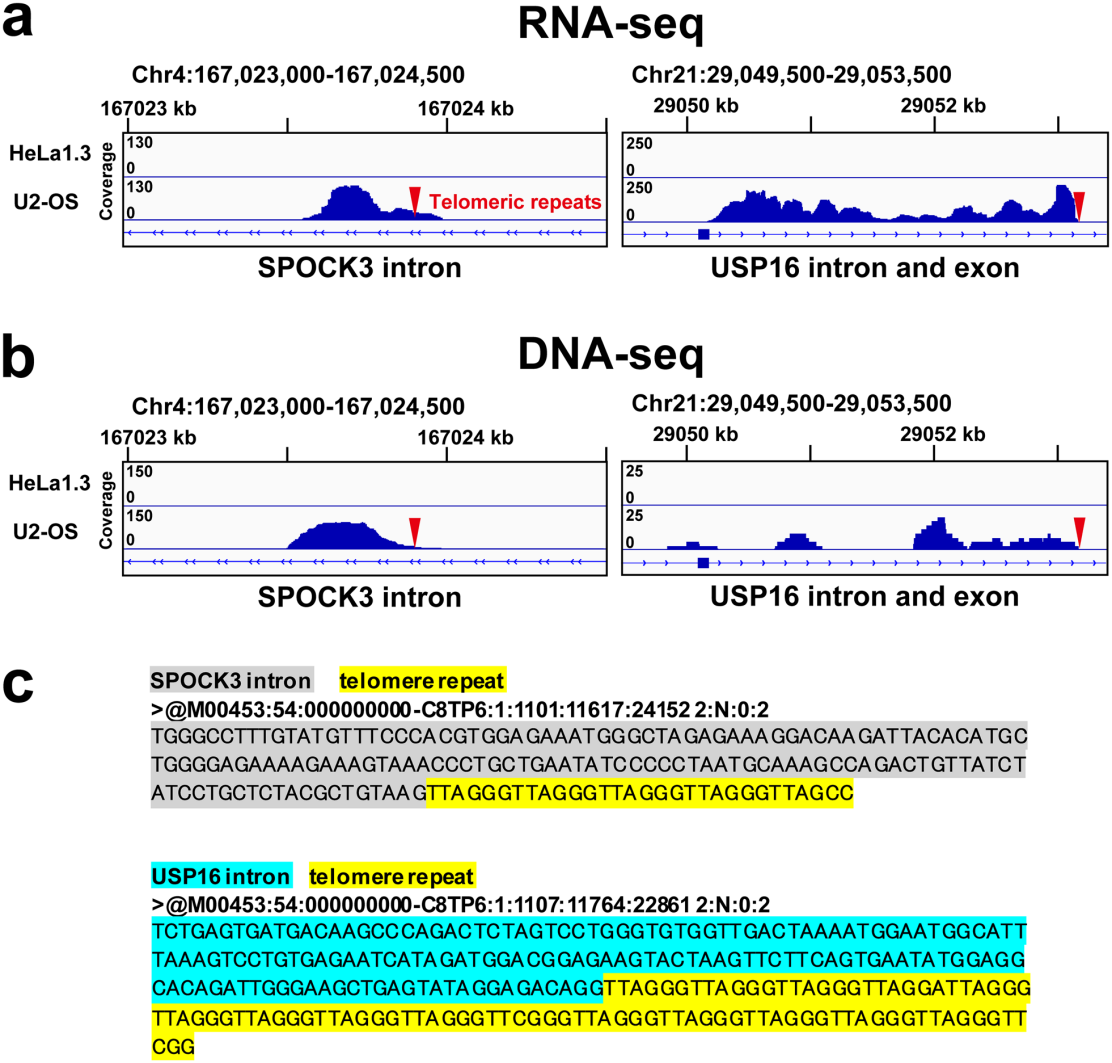
PI-PRICh detects ALT cell-specific ncRNAs transcribed from around the inserted telomeric repeats. **a** ncRNAs mapped to the introns of SPOCK3 and USP16 genes in ALT cells. These ncRNAs were highly enriched in the TH59-DB fraction from ALT cells (lower, U2-OS), but not from telomerase-positive cells (upper, HeLa1.3). Red arrowheads indicate the positions of the inserted telomeric repeats. **b** Sequence analysis of DNA fragments captured by TH59-DB. Genomic regions coding the introns of SPOCK3 and USP16 were specifically enriched in TH59-DB pull-down fraction of U2-OS, but not in that of HeLa1.3. The positions of the inserted telomeric repeats are indicated by red arrowheads. **c** Representative sequence reads containing both telomeric repeats (yellow) and SPOCK3 (gray) or USP16 intron region (light blue) in TH59-DB pull-down fractions from U2-OS cells

To search novel ncRNAs enriched in the pull-down chromatin from ALT cells, we used three filtering criteria: (1) the fragments per kilobase of per million mapped reads (FPKM) value, (2) the fold enrichment over total RNA of input sample before the telomeric purification, and (3) the relative ratio of ncRNAs between U2-OS cells and HeLa1.3 cells. Then, we identified some intronic ncRNAs (SPOCK3 and USP16 gene) with more than 100 FPKM enriched more than 25-fold, which were detected in U2-OS cells 50 times more than in HeLa1.3 cells (Fig. 5a and Additional file 9: Table S3 and Additional file 10: Table S4, Additional file 11: Table S5, Additional file 12: Table S6, Additional file 13: Table S7). On the other hand, TERC was the only ncRNAs enriched in HeLa1.3 cells based on these criteria.

### Identification of DNA fragments associated with telomeric repeats in ALT

As described above, telomere elongation by ALT involves homologous recombination between telomeres and also between non-telomeric regions and telomeres [48–50]. The latter can induce telomeric repeat insertions over the genome [49, 50]. Therefore, we wondered whether the identified intronic RNAs in ALT cells might be generated by transcription in the intron regions with inserted telomeric repeats. We found that the intron regions in ALT cells have insertions of telomeric repeats using genomic polymerase chain reaction (PCR) with various primer sets: one primer annealing to the intron regions and the other to (CCCTAA)_4_ or (TTAGGG)_4_ (Additional file 3: Fig. S3). The products were validated by Sanger sequencing.

To further confirm insertions of these telomeric repeats in intron regions, we analyzed DNA fragments captured by PI-PRICh. These intron regions were highly enriched in the TH59-DB pull-down fraction from U2-OS cells (Fig. 5b). We found chimeric sequence reads of the intron DNA sequence with (TTAGGG)n repeats, indicating that telomeric repeats were inserted into the intron regions (Fig. 5c).

In addition to ncRNAs from SPOCK3 and USP16 genes, we identified four other intronic ncRNAs corresponding to CKS1B, NRDC, PAM, and KIAA1671 genes (Additional file 4: Fig. S4 and Additional file 9: Table S3). Using similar analyses, we found that they also have flanking telomeric repeats (Additional file 3: Fig. S3, Additional file 5: Figure S5).

### Co-localization of ncRNAs at telomeres

To better understand the biological implications of the intronic ncRNAs identified, we examined localizations of the four intronic ncRNAs (USP16, SPOCK3, CKS1B, NRDC genes) in the U2-OS cell line by RNA-FISH (fluorescence in situ hybridization) analysis. Telomeres were simultaneously stained using the fluorescent TH59 [19, 20, 25]. As shown in Additional file 6: Fig. S6a and b, clear signals of USP16-intronic RNA were detected. Interestingly, some of them were co-localized with the telomere signals: 23% of USP16-positive cells, 50% of SPOCK3, 25% of CKS1B and 17% of NRDC-positive cells (Additional file 6: Fig. S6c). These results indicate that the intronic RNAs form foci and can localize near telomeres in the cell nuclei.

## Discussion

We demonstrated that PI-PRICh was reproducibly able to purify both proteins and RNAs associating with telomeric chromatin, whereas previous methods could only identify protein composition [13, 15]. Using PI-PRICh, we succeeded in identifying the known three classes of ncRNAs associated with telomeric chromatin with > 100-fold enrichment from MEL cells. The first class is a trans-acting ncRNA bound as a part of a ribonucleoprotein telomerase complex (i.e., TERC). The second class is a cis-acting ncRNA that functions as a major modulator of telomere maintenance (i.e., TERRA). Lastly, we identified ncRNAs associated as nascent transcripts from subtelomeric regions and interstitial telomeric sequences (third class). The comprehensive identification of ncRNAs with high enrichment relied on the unique binding mode of PI polyamide to target sequences of double-stranded DNA through minor grooves (Additional file 1: Fig. S1b) so that PI polyamide has almost no affinity to single-stranded and double-stranded RNA [53]. This property reduces contamination of abundant messenger RNAs and ribosomal RNAs during telomeric chromatin isolation by PI-PRICh, whereas chromatin isolation using other nucleic acid probes can potentially have abundant contaminant RNAs, because the nucleic acid probes often nonspecifically hybridize RNAs by mismatch pairing.

PI-PRICh is distinct from recently published methods for studying a genome-wide RNA–DNA contact, including MARGI, GRID-seq, ChAR-seq and RADICL-seq [54–57]. Chromatin-associated RNAs are immobilized to DNA by proximity ligation after restriction digestion, forming RNA–DNA chimeric sequences for sequencing with these techniques. However, obtained global RNA–chromatin interactomes must exclude the repetitive sequence regions due to a lack of restriction recognition sites. PI-PRICh is thus an excellent approach for the comprehensive identification of chromatin-associated ncRNAs found on repetitive sequences, which occupy approximately 45% of the human genome [58]. PI-PRICh can also be applied to other repetitive sequences, such as found in centromeres, to identify new chromatin-associated RNAs.

We also identified novel ncRNAs derived from the introns of gene loci in ALT cells. The intron regions (SPOCK3, USP16, CKS1B, NRDC, KIAA1671 and PAM) commonly included short telomeric repeats. DNA-seq showed that intronic DNA fragments were also enriched in the pull-down fraction from U2-OS cells. TH59-DB bound to inserted telomeric repeats in the PI-PRICh experiment and pulled down those ncRNAs nascently transcribed by RNA polymerase II. These data suggest that PI-PRICh can isolate DNA regulatory sequences with their associated RNAs from not only abundant repetitive sequence regions, but also single loci in the future.

Lastly, it would be intriguing to discuss possible functions of identified novel ncRNAs from the introns of several genes in the ALT cells. These intron regions (SPOCK3, USP16, CKS1B, NRDC, PAM, and KIAA1671) have short telomeric repeats. Interestingly, some of them seem to be associated with telomeres (Additional file 6: Fig. S6). This finding suggests that certain genome regions, including inserted telomeric repeats, are somehow tethered to telomeres in ALT cells. Such clustering might be facilitated by ncRNA and/or other proteins such as orphan nuclear receptors [49] (Additional file 6: Fig. S6d) and involve their high recombinogenic property in ALT cells.

With the evolution of new technologies, including cutting-edge imaging, advanced genomics and computational modeling, our knowledge of chromatin organization and dynamics is drastically increasing [1, 59, 60]. Our sensitive telomere labeling and capturing method can be performed under mild conditions, which opens the gate for other intriguing applications like super-resolution imaging of telomeres and the structural analysis of telomeric chromatin by cryo-electron microscopy without harsh treatments. Combining these techniques could help to elucidate how telomeric chromatin is organized in interphase cell nuclei and mitotic chromosomes. Therefore, telomere visualization and isolation using PI polyamide-based approaches discussed here would expand telomere biology and related medical science.

## Conclusion

PI-PRICh can simultaneously identify both the protein and RNA components of telomeric chromatin when directed against telomeric sequences. PI polyamide is thus a promising alternative for sequence-specific isolation of native chromatin regions with their associated proteins and RNAs, which will promote an increased understanding of chromatin organization and functions within the cell.

## Methods

### TH59-DB probe synthesis

Telomere PI polyamide TH59 containing a long spacer arm (24 PEG) (Additional file 1: Fig. S1a) was synthesized using the solid-phase synthetic method as described in the references [19, 20, 22]. ESI–TOF–MS *m/z* calculated for C_153_H_232_N_42_O_45_^4+^ [*M*+4H]^4+^ 844.4284 found 844.4276. For chromatin affinity purification, tandem hairpin TH59 was conjugated with desthiobiotin using EZ-Link™ NHS-desthiobiotin (16129, Thermo) to obtain the PI polyamide probe TH59-DB (Fig. 1b and Additional file 1: Fig. S1a) as described in the reference [23]. ESI–TOF–MS *m/z* calculated for C_163_H_249_N_44_O_47_^5+^ [*M* + 5H]^5+^ 714.9684 found 714.9686. TH59-DB was resuspended in *N*, *N*-dimethylformamide (DMF) (043-32361, Wako) and kept in the freezer until use.

### Telomere staining with TH59-DB

HeLa1.3 cells were maintained at 37 °C (5% CO_2_) in DMEM (D5796, Sigma) containing 10% FBS. For polyamide staining, cells were grown on coverslips coated with poly-lysine. The cell coverslips were washed twice in phosphate-buffered saline (PBS) and fixed with 1.85% formaldehyde in PBS for 15 min at room temperature. For blocking, cells were treated with 10% normal goat serum (NGS) (S26-100, Millipore) in TE buffer (10 mM Tris–HCl pH 7.5, 1 mM EDTA) for 30 min at room temperature. After a brief rinse with TE buffer, the cells were incubated with 10% NGS, 100 nM TH59-DB in DMF, mouse anti-TRF2 (ab13579, Abcam), and 0.5 μg/mL DAPI (4′,6-diamidino-2-phenylindole) (10236276001, Roche) in TE buffer at 37 °C for 1 h. After washing with TEN200 buffer (10 mM Tris–HCl, pH7.5, 1 mM EDTA, and 200 mM NaCl), cells were incubated with 10% NGS, streptavidin Alexa488 (s11223, Invitrogen) and anti-mouse Alexa594 (AA11032, Invitrogen) in TE for 1 h at room temperature. The cells on the coverslips were washed with TEN200 buffer (five times for 3 min), then mounted. Cell images were recorded with a DeltaVision microscope and deconvolved to eliminate out-of-focus blur to obtain clearer pictures. The deconvolved images were projected (‘Quick Projection’ tool) to obtain the maximum intensity of telomere signals.

### Plasmid pull-down assay

This assay was performed as described [14]. For each assay, 200 ng linearized plasmids (telomeric repeats containing plasmid and empty vector) were resuspended in LB3JD buffer (10 mM HEPES–NaOH, pH 7.7, 100 mM NaCl, 2 mM EDTA, 1 mM EGTA, 0.2% SDS, 0.1% SLS) containing 0.5 μM TH59-DB. The mixture was incubated at 37 °C for 30 min. During incubation, 15 µl of MyOne C1 beads (DB6502, VERITAS) was added. After incubation at room temperature for 30 min with shaking, beads were immobilized on a magnetic stand. The supernatant was collected as flow-through. Beads were washed five times with 1 mL of LB3JD at room temperature, resuspended in LB3JD containing 12.5 mM D-biotin (B-20656, Invitrogen) and incubated at 65 °C for 15 min for elution. One-tenth volumes of the input, flow-through, and elution fraction were analyzed by agarose gel electrophoresis, followed by ethidium bromide (EtBr) staining. Band intensities were quantified relative to input by Fiji software in three independent experiments [61]. As a negative control for telomeric chromatin isolation, the masked TH59-DB was prepared as follows: TH59-DB was incubated with a double-stranded DNA oligonucleotide (TTAGGG/CCCTAA)_4_ for 60 min at 37 °C.

### Chromatin purification from mouse (MEL) cells by TH59-DB

MEL cells were grown in DMEM with 10% FBS and 2 mM l-glutamine. The telomere length of MEL cells is not available but could be between > 23 and < 100 kb based on telomere lengths described in mouse embryonic fibroblasts [62]. 2 L of MEL cells (~ 2–4 × 10^6^ cells/ mL) grown in a roller bottle were crosslinked with 3.7% formaldehyde in PBS for 30 min at room temperature. After washing four times with PBS, cells were transferred in sucrose buffer (0.3 M sucrose, 10 mM HEPES–NaOH, pH 7.7, 1% Triton X-100, 2 mM MgOAc) and lysed with 20 strokes of a Dounce homogenizer with a tight pestle. Chromatin was pelleted by centrifugation at 3200*g* for 10 min at 4 °C. The pellet was resuspended in the same volume of glycerol buffer (25% glycerol, 10 mM HEPES–NaOH, pH 7.9, 0.1 mM EDTA, 0.1 mM EGTA, 5 mM MgOAc), and then frozen in liquid nitrogen and stored at − 80 °C or used immediately for telomeric-chromatin-isolation. The chromatin pellet was washed with PBS five times and with LB3JD containing 1 mM phenylmethylsulphonyl fluoride (LB3JD-PMSF) (P7626, Sigma). After centrifugation at 3000*g* for 8 min, the pellet was resuspended in a 1.5 × volume of LB3JD-PMSF buffer and then passed three times through a French press (FA-078A, Thermo) at 25,000 p.s.i. at room temperature. The solubilized chromatin sample was collected at 20,000*g* for 15 min at 4 °C and was heated at 58 °C for 5 min. LB3JD-PMSF-pre-equilibrated streptavidin agarose beads (20361, Thermo) were added, and the sample was incubated at 4 °C overnight. The mixture was applied to Sephacryl S-400-HR spin columns (17-0609-10, Roche). The sample was centrifuged at 20,000*g* for 15 min and SDS was added to the supernatant to a final concentration of 0.2%. About 30 mg chromatin was incubated with 600 pmol of TH59-DB or masked TH59-DB for 2 h at 37 °C. The sample was then centrifuged at 20,000*g* for 15 min and then the supernatant was added to streptavidin-FG beads (TAS8848 N1170, Tamagawa-Seiki) pre-equilibrated with LB3JD. The sample was incubated at room temperature for 2 h on a nutator. Beads were washed with 10 mL of LB3JD five times at room temperature. Beads were collected in a 1.5 mL tube and additionally washed with shaking in LB3JD containing 30 mM NaCl at 42 °C for 10 min and then in LB3JD containing 10 mM NaCl at 42 °C for 10 min. Beads were resuspended in LB3JD containing 12.5 mM D-biotin and incubated at 37 °C for 1 h for elution with shaking. Cleared supernatant was collected as eluate and kept at − 80 °C until further analysis. PICh protocol was performed as previously described with the LNA probe hybridizing telomere sequence [13]. Briefly, the chromatin sample preparation from MEL cells was done as described for PI-PRICh. About 30 mg chromatin was hybridized to 1500 pmol of LNA probe for the telomeric repeat and the scramble LNA probe: desthiobiotin-PEG24- 5′-TtAgGgTtAgGgTtAgGgTtAgGgt-3′ and desthiobiotin-PEG24- 5′-GaTgTgTgGaTgTggAtGtGgAtgTgg-3′, respectively, where capitalized letters are LNA residues and small letters are DNA residues. Hybridization was performed by sequential incubations at 25 °C for 3 min, 72 °C for 7 min, and 37 °C for 3 h. The sample was then centrifuged at 20,000*g* for 15 min, and the supernatant was added to MyOne C1 streptavidin beads (DB65002, Thermo) pre-equilibrated with LB3JD. The subsequent procedures, such as wash and elution steps, were performed as described for chromatin purification by TH59-DB.

### Chromatin sample preparation from adherent cells for purification with TH59-DB

HeLa1.3 and U2-OS cells (~ 10^9^ cells) were grown in DMEM with 10% FBS. Telomere lengths of U2-OS cells and HeLa1.3 cells were reported to be > 30 kb and > 20 kb, respectively [63, 64]. The cells were washed with PBS and crosslinked with 3.7% formaldehyde in PBS for 30 min at room temperature. After being washed twice with cold PBS, cells were scraped with a scraping buffer (0.05% Tween-20 in PBS). The following procedures are the same as chromatin preparation of MEL cells described above.

### Protein extraction for mass spectrometry

For protein analysis of the eluate, trichloroacetic acid (18% final) precipitation was performed, and the pellet was incubated with 80 mL crosslinking reversal solution (250 mM Tris–HCl (pH 8.8), 2% SDS, 1 M 2-mercaptoethanol) at 99ºC for 30 min. Proteins from the eluate from PI-PRICh with masked TH59-DB or TH59-DB probes were separated using a 12% Bis–Tris acrylamide pre-cast gel (NP0343BOX, Invitrogen). The gels were stained with colloidal blue for MS analysis or silver-stained (Silver Quest kit) (LC6070, Invitrogen) for detecting unique bands purified by PI-PRICh using the TH59-DB probe or subjected to western blot analyses with an anti-TRF1 antibody (kind gift from Y. Shinkai). For comprehensive protein identifications, gel lanes were cut into regions according to the banding pattern and subjected to MS analysis (NIG Mass Spectrometry Facility).

### RNA extraction and preparation of cDNA libraries for next-generation sequencing

To extract total RNA, > 2.5 mg isolated chromatin was incubated in de-crosslinking buffer (32 mM Tris–HCl pH 8.0, 320 μg/mL Protease K, 0.8% SDS) for 6 h at 65 °C. An equal volume of acidic phenol was added, then vortexed, and incubated for 5 min at room temperature. After adding an equal volume of chloroform: isoamyl alcohol (24:1, v/v) and centrifuging at 16,000*g* for 5 min at 4 °C, the upper aqueous phase was transferred to a new tube. This step was repeated. One-tenth volume of 3 M sodium acetate, glycogen and double volume of ethanol were added and incubated overnight at − 80 °C. After centrifugation at 20,000*g* for 30 min at 4 °C, the pellet was retained, washed with 70% ethanol and dried up. The dried pellet was dissolved in diethylpyrocarbonate (DEPC)-treated water (36415-54, Nakarai) and kept at − 80 °C until use. Total RNA content of each sample was measured using Qubit (Q32851, Thermo), and the quality of RNA samples was assessed by an Agilent 2100 Bioanalyzer using Agilent RNA 6000 pico kit (5067-1513, Agilent). cDNA libraries were synthesized by the SMARTer Stranded Total RNA-Seq Kit v2 (634411, Takara). The size distributions of the libraries were checked by an Agilent 2100 Bioanalyzer using an Agilent High Sensitivity DNA kit (5067-4626, Agilent). Pooled amplicon library was sequenced with paired-end 2 × 150 bp reads on the Illumina MiSeq platform.

### Detection of TERRA/ARRET in the RNA-Seq data and DNA-Seq data

To estimate the number of putative TERRAs, reads containing (TTAGGG)_5_ or (CCCTAA)_5_ repeats were extracted from each Read 2 (R2) fastq file using the grep command in the UNIX system.

### Data processing and software for RNA-seq

First, adapter sequences were removed. The reads were trimmed for low quality with CUTADAPT and PRINSEQ using a composite set of Illumina adapters, a minimum quality score of 20 and a minimum length of 25 [65, 66]. Filtered mouse and human sequence data were aligned to the mouse mm10 genome and human hg38 genome, respectively, using HISAT2 [67]. To find telomere-enriched ncRNAs, the mapped fragments in TH59-DB pulled-down fractions were assembled into RNA transcripts and annotated using Cufflinks software [68]. The amounts of RNA transcripts were compared based on fragments per kilobase of per million mapped reads (FPKM) between samples using featureCounts and R [69, 70]. RNA-seq data were also visualized by igvtools [71].

### Mapping of TERRA in MEL cells

For mapping of mouse TERRA, we focused on a 30-kb region adjacent to the telomere repeat of each long (q) arm of the chromosome [43]. Centromere-adjacent telomeres on short (p) arms were not analyzed, because they were not sequenced. Using featureCounts, FPKM values of the subtelomeric regions (Additional file 14: Table S8) were compared between input and TH59-DB pull-down fractions.

### Mapping of TERRA in HeLa1.3 and U2-OS

For mapping human TERRA on the X/Y chromosome, custom gene annotation containing the sequence of TelBam3.4 [51, 52] was generated for quantification of telomere-associated TERRA. Individual reads of RNA-Seq data were mapped to the custom gene and human genome hg38 by Bowtie2 with default settings except for extraction of uniquely mapped reads, respectively [72]. Mapped reads were assembled into transcripts and annotated by Cufflinks. The number of reads overlapped to a region downstream of the transcription start site in TelBam3.4 were counted by featureCounts and normalized as reads per kilobase of exon model per million mapped reads (RPKM) on TelBam3.4 and human genome hg38.

### DNA extraction and preparation of cDNA libraries for next-generation sequencing

To extract DNA, the pull-down chromatin fractions were incubated in de-crosslinking buffer (50 mM Tris–HCl pH 8.0, 200 μg/mL Protease K, 2% SDS) overnight at 65 °C. An equal volume of phenol: chloroform: isoamyl alcohol (25:24:1,v/v) (311-90151, Wako) was added, vortexed and then centrifuge at 16,000*g* for 5 min at room temperature. The upper aqueous phase was transferred to a new tube. This step was repeated. One-tenth volume of 3 M sodium acetate, glycogen, and the double volume of ethanol were added and incubated for 2 h at − 80 °C. After centrifugation at 20,000*g* for 30 min at 4 °C, the pellet was retained, washed with 70% ethanol and dried. The dried pellet was dissolved in 10 μg/mL RNase A in DNA dilution buffer attached to DNA SMART ChIP-Seq Kit (634865, Takara). After incubating for 1 h at 37 °C, DNA was purified by phenol–chloroform extraction and ethanol precipitation to remove RNase A. DNA samples were kept at − 30 °C until use. DNA concentrations of each sample were measured by Qubit. cDNA libraries were synthesized by DNA SMART ChIP-Seq Kit (634865, Takara). The size distributions of the libraries were checked by an Agilent 2100 Bioanalyzer using Agilent High Sensitivity DNA kit. Pooled amplicon library was sequenced on the Illumina MiSeq platform with paired-end 2 × 250 bp reads for HeLa1.3 and U2-OS cells, and with single-end 75 bp reads for MEL cells.

### Detection of ncRNA-associated telomeric repeats in the DNA-seq

To confirm telomeric repeats were inserted near the genomic region, where ncRNA was transcribed, sequence reads in the DNA-seq containing ncRNA sequences were extracted from each fastq file using the grep command in the UNIX system. The sequences for the grep command are shown in Additional file 15: Table S9.

### Data processing and software for DNA-seq

First, adapter sequences were removed. The reads were trimmed for low quality with CUTADAPT and PRINSEQ, using a composite set of Illumina adapters, a minimum quality score of 20, and a minimum length of 25. Filtered human sequence data were aligned to the human hg38 genome using Bowtie2. DNA-seq data were also visualized by igvtools.

### Genomic PCR to test telomeric sequence insertion

To purify genomic DNA, cells grown on φ10 cm dishes were collected and incubated in lysis buffer (10 mM Tris–HCl pH 8.0, 0.1 M EDTA, 0.5% SDS, 20 μg/mL RNase A) for 1 h at 37 °C. After adding 100 μg/mL Proteinase K (10432, Wako), the cell lysate was incubated for 3 h at 50 °C. DNA was isolated using phenol–chloroform extraction and ethanol precipitation. To test if the telomeric repeat sequence located to the flanking region of the introns, PCR reactions with primer sets shown in Additional file 15: Table S9 were performed on genomic DNA from HeLa1.3 and U2-OS cells using the KOD-FX (KFX-101, Toyobo). PCR products were analyzed by agarose electrophoresis, followed by ethidium bromide staining.

### RNA fluorescence in situ hybridization

Telomere staining with RNA-FISH was performed following an RNA-FISH method combined with immunofluorescence as described in [73]. Briefly, cells were seeded on poly-lysine coated coverslips and incubated overnight at 37 °C in 5% CO_2_. Coverslips were washed with PBS and fixed with 3.7% formaldehyde in PBS at room temperature for 10 min. After 5 min washing with PBS, cells were permeabilized with 0.1% Triton X-100 in PBS for 10 min and then washed with PBS for 5 min. The coverslips were incubated with prehybridization solution [2 × SSC, 1 × Denhards solution, 50% (v/v) formamide, 10 mM EDTA pH 8.0, 100 μg/mL yeast tRNA and 0.01% Tween-20] at 55 °C for 1 h. The prehybridized coverslips were then incubated with hybridization solution [2 × SSC, 1 × Denhards solution, 50% (v/v) formamide, 10 mM EDTA pH 8.0, 100 μg/mL yeast tRNA, 0.01% Tween-20, 5% (w/v), dextran sulfate and DIG-labeled RNA probes, which were generated using DIG RNA Labeling Kit (11277073910, Roche)] at 55 °C overnight. After hybridization, the coverslips were washed twice with wash buffer [2 × SSC, 50% (v/v) formamide and 0.01% Tween-20] at 55 °C for 30 min twice. To remove non-specific binding of RNA probe, cells were incubated in RNase A buffer (1 μg/mL RNase A, 0.5 M NaCl, 10 mM Tris–HCl pH 8.0, 1 mM EDTA) for 1 h at 37 °C. After RNase A treatment, cells were washed with wash buffer2 (2 × SSC, 0.01% Tween-20) and wash buffer3 (0.2 × SSC, 0.01% Tween-20) for 30 min at 55 °C. For detection, coverslips were washed in TBST (Tris-buffered saline solution containing 0.01% Tween-20) for 5 min at room temperature, incubated with blocking solution [1 × Blocking reagent (11096176001, Roche) in TBST] for 5 min at room temperature, and then incubated with anti-DIG Rhodamine (11207750910, Roche) and 15 nM Silicon Rhodamine (SiR)-TH59 for 90 min at room temperature. After staining with 0.5 μg/mL DAPI in TBST for 5 min at room temperature, unbound antibodies were removed by washing three times with TBST for 5 min. Coverslips were then mounted and image acquisition was performed with a DeltaVision microscope. Signal intensities were measured by Plot Profile tools in Fiji and line plots were created using R software [70].

## Supplementary Information

**Additional file 1: Figure S1.** Structure of TH59-DB and enrichment of ncRNAs by PI-PRICh within subtelomeric regions of MEL cells. **a** Chemical structure of TH59-DB. Py, N-methyl pyrrole (blue) and Im, N-methyl imidazole (red). **b** A structural model of TH59-DB binding to DNA. **c**, ncRNAs mapped to the region near the end of each chromosome (chromosomes 13, 14, 15, 16, 17, 18) from Experiment 1. A genomic view of the terminal regions of the q arm of each chromosome based on the mouse reference genome, GRCm38 (mm10). RNAs were mapped to the terminal region of each chromosome. Results with the input, masked TH59-DB and TH59-DB are shown.

**Additional file 2: Figure S2.** Comprehensive identification of telomeric chromatin-associated RNAs in MEL cells (Experiment 2). **a** The percentage (yellow) of telomeric repeat reads, including TERRA transcripts, in the input and TH59-DB pull-down fractions from MEL cells. The number of single reads, including (TTAGGG)_5_ or (CCCTAA)_5_, extracted from input and TH59-DB pull-down fractions were divided by the total number of reads. The gray color depicts other reads. **b** Scatter plot of fragments per kilobase of per million mapped reads (FPKM) of the TH59-DB pull-down fraction versus that of the input sample for each RNA. The telomerase RNA component, TERC (most enriched in the TH59-DB pull-down fraction), is highlighted in blue. RNAs enriched more than 100-fold in the TH59-DB pull-down fraction are plotted below a red dotted line. **c** Bar graph of FPKM of telomerase RNA-component (TERC) in input and TH59-DB pull-down fractions. **d** Sequence analysis of DNA fragments captured by TH59-DB telomeric repeat reads. The percentage (yellow) of telomeric repeat reads including TTAGGG or CCCTAA, in the input and TH59-DB pull-down fractions from MEL cells. Note that results are similar to those shown in Fig. 3.

**Additional file 3: Figure S3.** Genomic PCR to test telomeric sequence insertion. **a** A scheme of genomic PCR to test telomeric repeat insertion into the flanking regions of SPOCK3, USP16, CKS1B, NRDC, PAM and KIAA1671 introns. Primer sets and expected PCR products are shown. **b** PCR products of each intron amplified by the three primer sets with HeLa1.3 and ALT U2-OS genomic DNA. Lanes 1–3 and lanes 5–7 for SPOCK3, lanes 8–10, and lanes 12–14 for USP16, lanes 15–17 and lanes 19–21 for CKS1B, lanes 22–24 and lanes 26–28 for NRDC, lanes 29–31 and lanes 33–35 for PAM, lanes 36–38 and lanes 40–42 for KIAA1671. 100 bp or 1 kb ladder was loaded at the center lane of the gel as a size marker (M).

**Additional file 4: Figure S4.** PI-PRICh identifies ALT cell-specific ncRNAs transcribed from around the inserted telomeric repeats. Telomeric repeat-associated ncRNAs mapped to intron regions of CKS1B, NRDC, PAM and KIAA1671 genes in ALT U2-OS cells. These ncRNAs were highly enriched in the TH59-DB pull-down fraction of U2-OS cells, but not in HeLa1.3. The positions of the inserted telomeric repeats are indicated by red arrowheads.

**Additional file 5: Figure S5.** DNA sequence analysis of TH59-DB binding sites. Genomic regions coding the introns of CKS1B, NRDC, PAM, and KIAA1671 were specifically enriched in the TH59-DB pull-down fraction from ALT U2-OS cells. These intron regions were enriched in the TH59-DB fraction from U2-OS, but not in HeLa1.3 cells. The positions of the inserted telomeric repeats are indicated by red arrowheads.

**Additional file 6: Figure S6**. RNA-FISH for intronic ncRNAs identified by PI-PRICh in ALT cells and a model for chromatin tethering to telomeres with ncRNAs. **a** RNA-FISH for the USP16 intron and the simultaneous telomere labeling with the fluorescent TH59 probe. First column, DAPI signal; second column, ncRNA signal of TERRA; third column, fluorescent TH59 signal (telomere); fourth column, merged image of DAPI (blue), ncRNA (green) and telomere (red). **b** Enlarged images of the boxed region in **a**. Line plot of the USP16-intronic RNA signal and the telomeric signal on the white dotted line in the left merged image. **c** Bar graph of percentages of cells in which USP16, SPOCK3, CKS1B and NRDC intronic RNA signals were co-localized with telomere signal. Three independent experiments were performed and the numbers of ncRNA signals measured for U2-OS cells were from 100 cells for each experiment. Error bars show standard deviation. **d** A model of how the genome region with inserted telomeric repeats tethers to telomere in ALT cells.

**Additional file 7: Table S1.** Mass spectrometry data of PI-PRICh experiments from MEL cells.

**Additional file 8: Table S2.** Mapped and counted RNA fragments from MEL cells. Sheet 1: RNAs with over 100-fold enrichment in TH59-DB fraction by PRICh in MEL experiment 1. Sheet 2: A non-filtered result of counted RNA fragments in MEL experiment 1. Sheet 3: RNAs with over 100-fold enrichment in TH59-DB fraction by PRICh in MEL experiment 2. Sheet 4: A non-filtered result of counted RNA fragments in MEL experiment 2.

**Additional file 9: Table S3.** RNAs enriched in the U2-OS or HeLa1.3 cell line by filtering criteria. Sheet 1: U2-OS enriched RNAs by filtering criteria. Sheet 2: HeLa1.3 enriched RNAs by filtering criteria.

**Additional file 10: Tables S4.** Filtration procedure to identify RNAs enriched in the U2-OS or HeLa1.3 cell line. RNAs in U2-OS experiment 1. Sheet 1: Non-filtrated count data. Sheet 2: Enriched more than 25-fold. Sheet 3: 50 times more than another cell line.

**Additional file 11: Tables S5.** Filtration procedure to identify RNAs enriched in the U2-OS or HeLa1.3 cell line. RNAs in HeLa1.3 experiment 1. Sheet 1: Non-filtrated count data. Sheet 2: Enriched more than 25-fold. Sheet 3: 50 times more than another cell line.

**Additional file 12: Table S6.** Filtration procedure to identify RNAs enriched in the U2-OS or HeLa1.3 cell line. RNAs in U2-OS experiment 2. Sheet 1: Non-filtrated count data. Sheet 2: Enriched more than 25-fold. Sheet 3: 50 times more than another cell line.

**Additional file 13: Table S7.** Filtration procedure to identify RNAs enriched in the U2-OS or HeLa1.3 cell line. RNAs in HeLa1.3 experiment 2. Sheet 1: Non-filtrated count data. Sheet 2: Enriched more than 25-fold. Sheet 3: 50 times more than another cell line.

**Additional file 14: Table S8.** Mouse subtelomeric and telomeric regions for FPKM calculation in Fig. 3d.

**Additional file 15: Table S9.** Primers for telomeric insertion test and for RNA FISH and DNA sequences to search targets for the grep command in the UNIX system.

## Data Availability

PI-PRICh data are available in NCBI GEO database as GSE181609.
