# Supplementary figures and images for: Telomere-specific chromatin capture using a pyrrole–imidazole polyamide probe for the identification of proteins and non-coding RNAs
Source: Epigenetics Chromatin. 2021 Oct 9;14:46. doi: 10.1186/s13072-021-00421-8 (PMC8502363; doi:10.1186/s13072-021-00421-8)

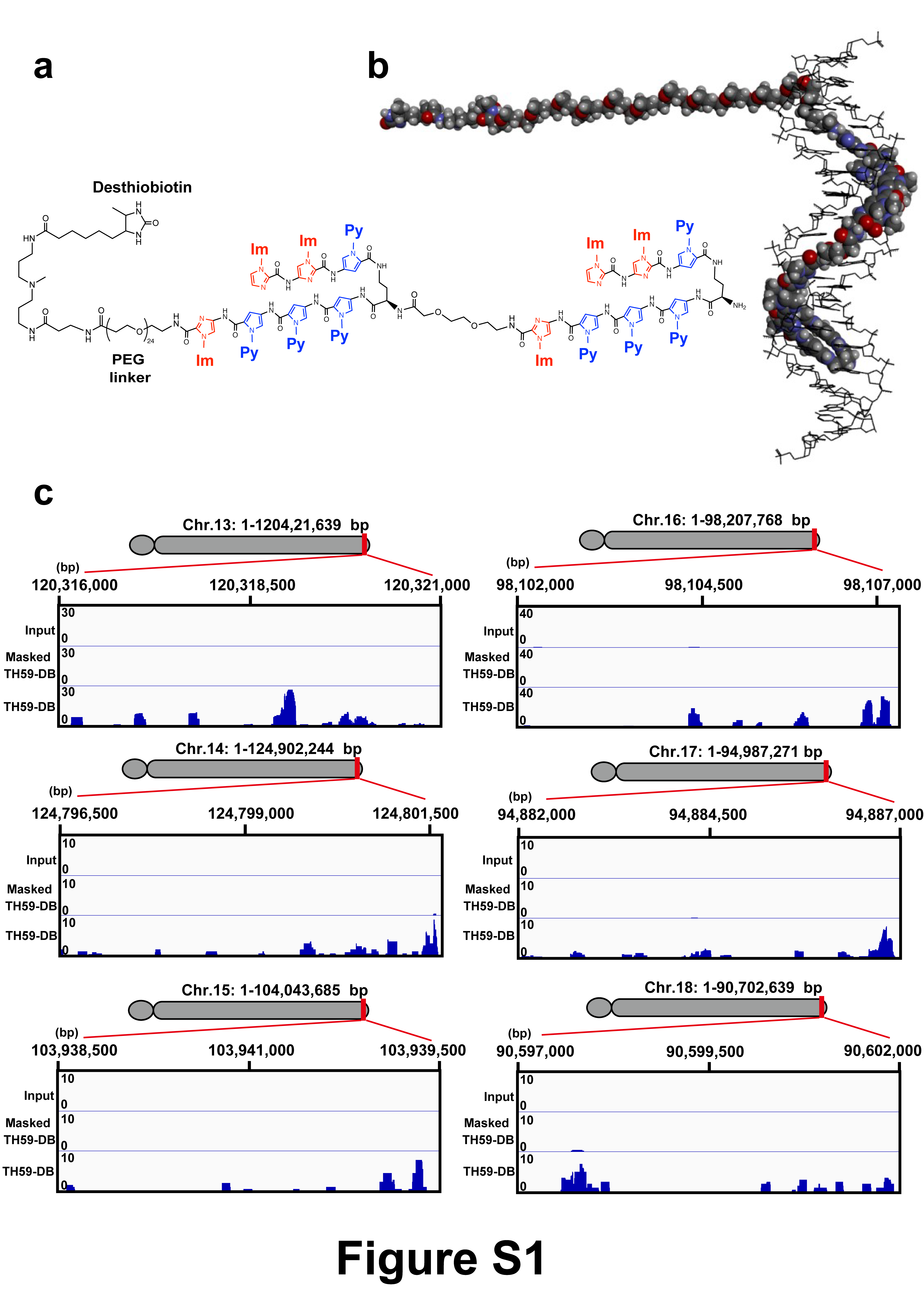

Supplement: Supplementary file 1 — Additional file 1: Figure S1. Structure of TH59-DB and enrichment of ncRNAs by PI-PRICh within subtelomeric regions of MEL cells. a Chemical structure of TH59-DB. Py, N-methyl pyrrole (blue) and Im, N-methyl imidazole (red). b A structural model of TH59-DB binding to DNA. c, ncRNAs mapped to the region near the end of each chromosome (chromosomes 13, 14, 15, 16, 17, 18) from Experiment 1. A genomic view of the terminal regions of the q arm of each chromosome based on the mouse reference genome, GRCm38 (mm10). RNAs were mapped to the terminal region of each chromosome. Results with the input, masked TH59-DB and TH59-DB are shown. [file 13072_2021_421_MOESM1_ESM.tif]

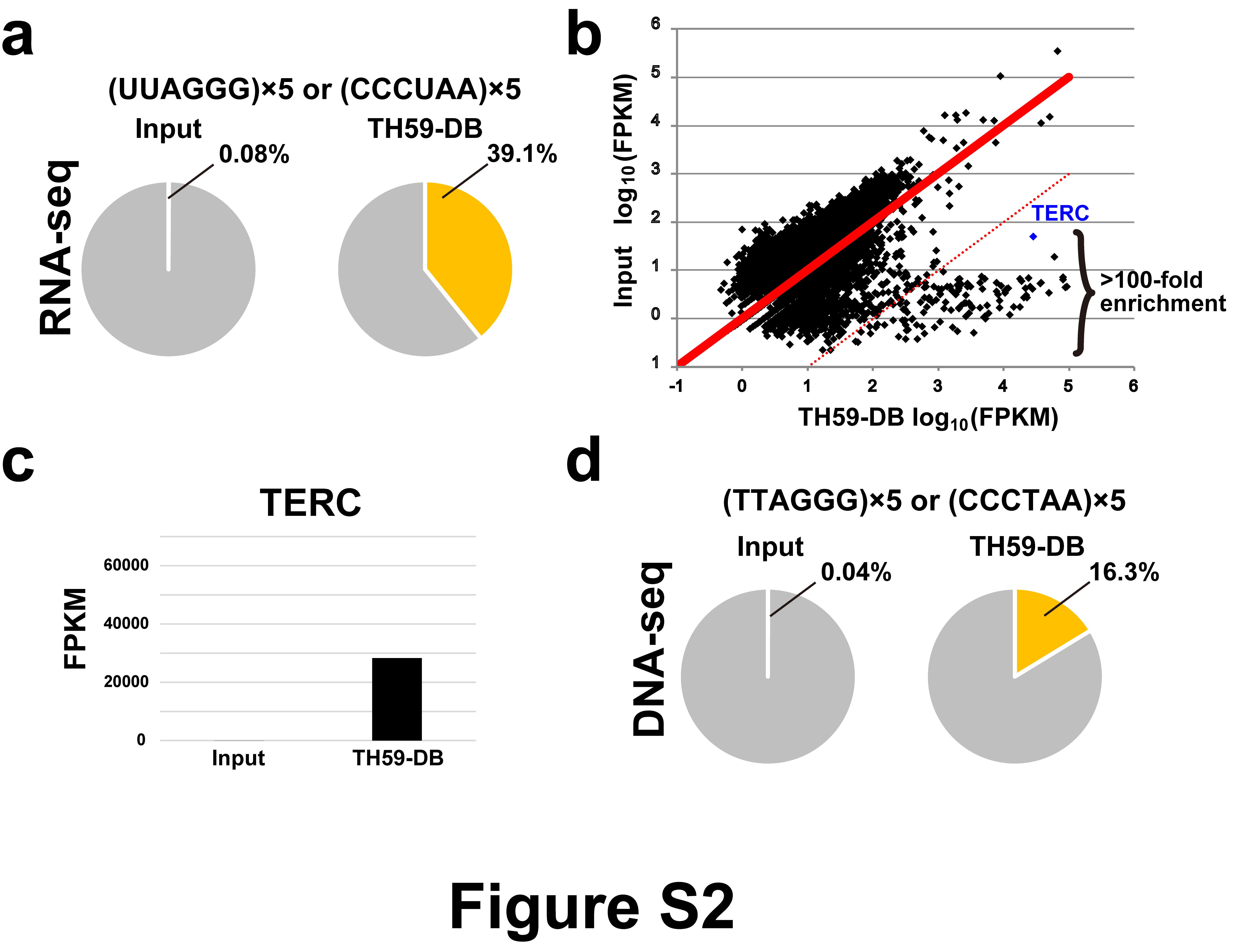

Supplement: Supplementary file 2 — Additional file 2: Figure S2. Comprehensive identification of telomeric chromatin-associated RNAs in MEL cells (Experiment 2). a The percentage (yellow) of telomeric repeat reads, including TERRA transcripts, in the input and TH59-DB pull-down fractions from MEL cells. The number of single reads, including (TTAGGG)5 or (CCCTAA)5, extracted from input and TH59-DB pull-down fractions were divided by the total number of reads. The gray color depicts other reads. b Scatter plot of fragments per kilobase of per million mapped reads (FPKM) of the TH59-DB pull-down fraction versus that of the input sample for each RNA. The telomerase RNA component, TERC (most enriched in the TH59-DB pull-down fraction), is highlighted in blue. RNAs enriched more than 100-fold in the TH59-DB pull-down fraction are plotted below a red dotted line. c Bar graph of FPKM of telomerase RNA-component (TERC) in input and TH59-DB pull-down fractions. d Sequence analysis of DNA fragments captured by TH59-DB telomeric repeat reads. The percentage (yellow) of telomeric repeat reads including TTAGGG or CCCTAA, in the input and TH59-DB pull-down fractions from MEL cells. Note that results are similar to those shown in Fig. 3. [file 13072_2021_421_MOESM2_ESM.tif]

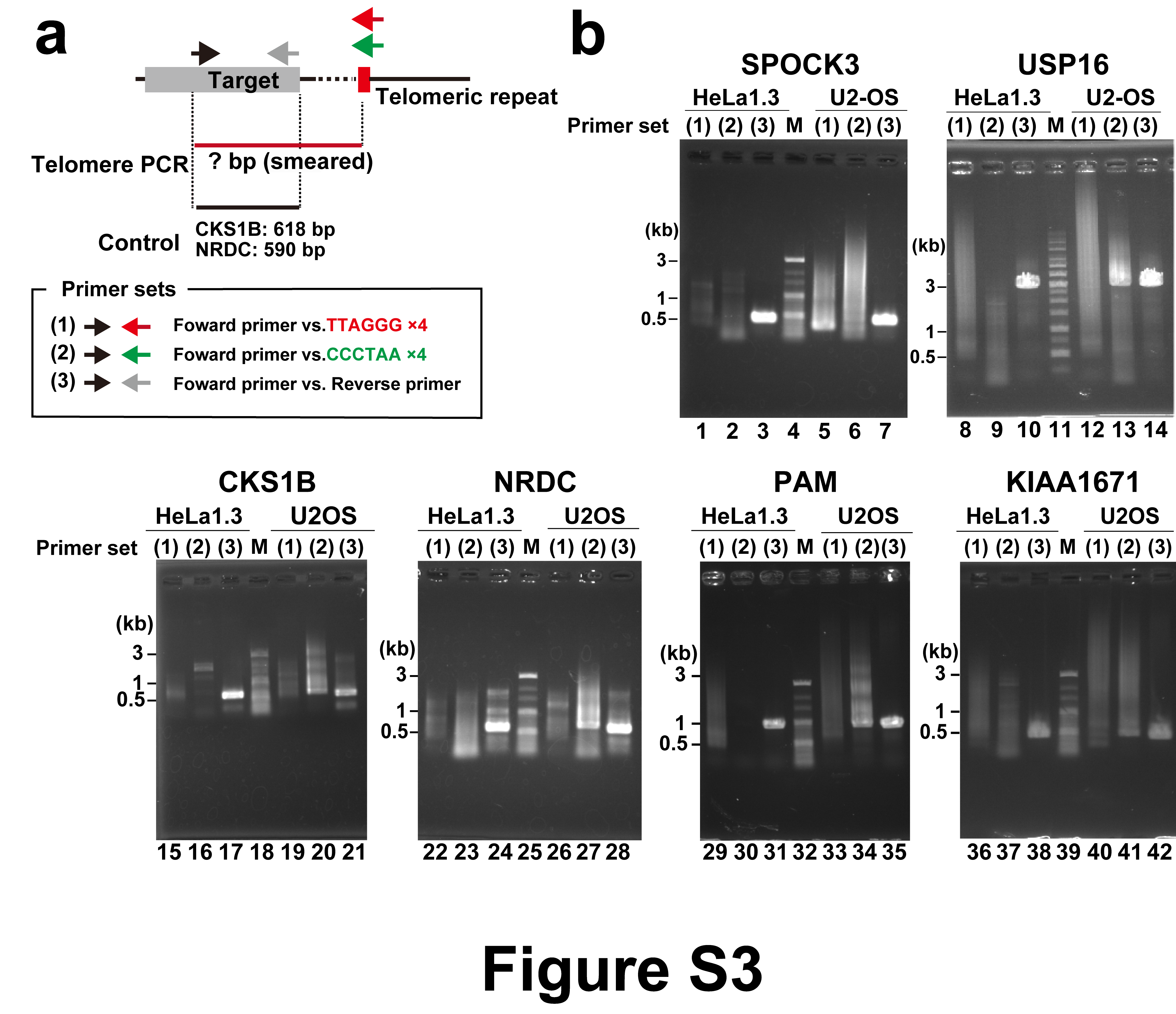

Supplement: Supplementary file 3 — Additional file 3: Figure S3. Genomic PCR to test telomeric sequence insertion. a A scheme of genomic PCR to test telomeric repeat insertion into the flanking regions of SPOCK3, USP16, CKS1B, NRDC, PAM and KIAA1671 introns. Primer sets and expected PCR products are shown. b PCR products of each intron amplified by the three primer sets with HeLa1.3 and ALT U2-OS genomic DNA. Lanes 1–3 and lanes 5–7 for SPOCK3, lanes 8–10, and lanes 12–14 for USP16, lanes 15–17 and lanes 19–21 for CKS1B, lanes 22–24 and lanes 26–28 for NRDC, lanes 29–31 and lanes 33–35 for PAM, lanes 36–38 and lanes 40–42 for KIAA1671. 100 bp or 1 kb ladder was loaded at the center lane of the gel as a size marker (M). [file 13072_2021_421_MOESM3_ESM.tif]

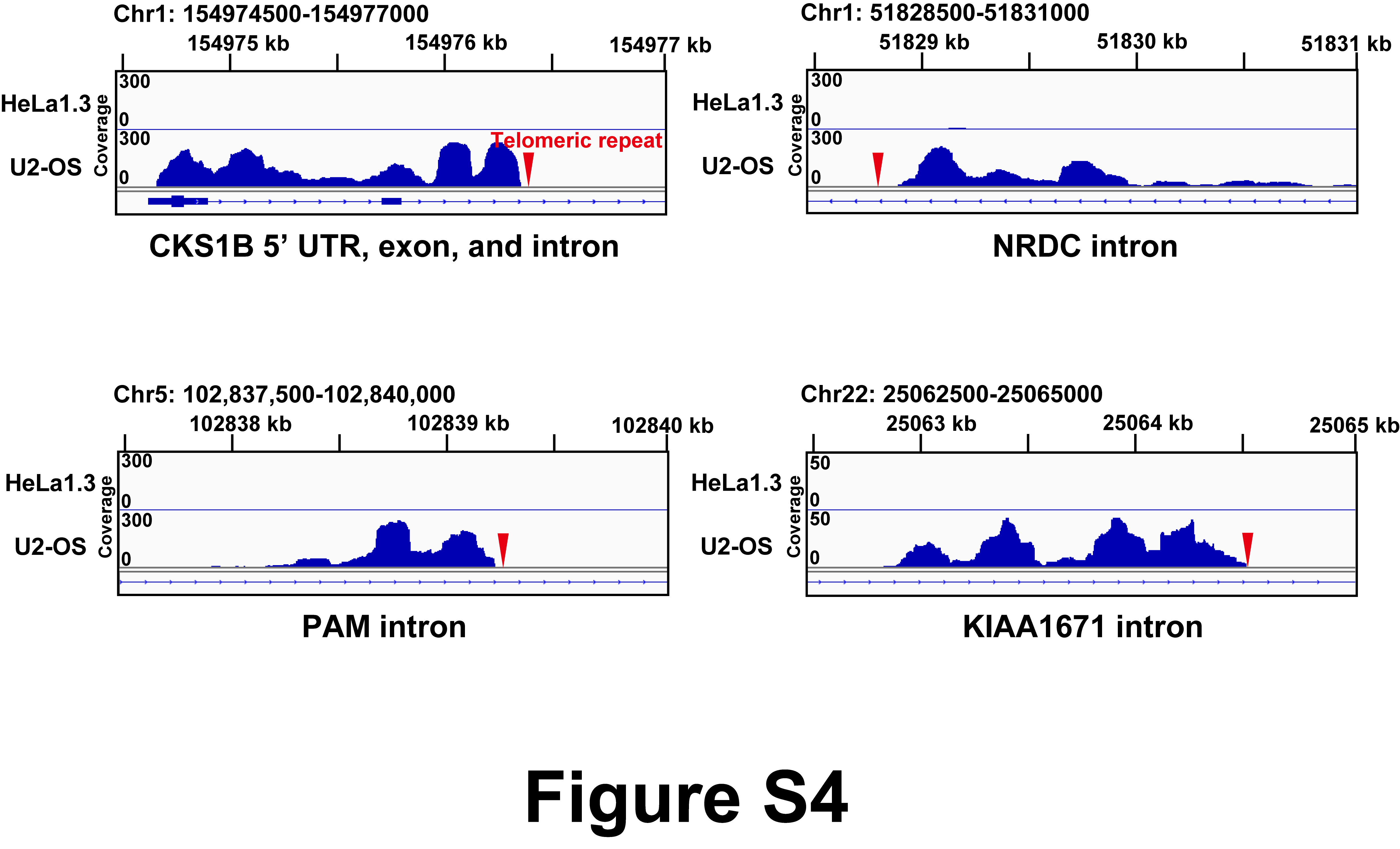

Supplement: Supplementary file 4 — Additional file 4: Figure S4. PI-PRICh identifies ALT cell-specific ncRNAs transcribed from around the inserted telomeric repeats. Telomeric repeat-associated ncRNAs mapped to intron regions of CKS1B, NRDC, PAM and KIAA1671 genes in ALT U2-OS cells. These ncRNAs were highly enriched in the TH59-DB pull-down fraction of U2-OS cells, but not in HeLa1.3. The positions of the inserted telomeric repeats are indicated by red arrowheads. [file 13072_2021_421_MOESM4_ESM.tif]

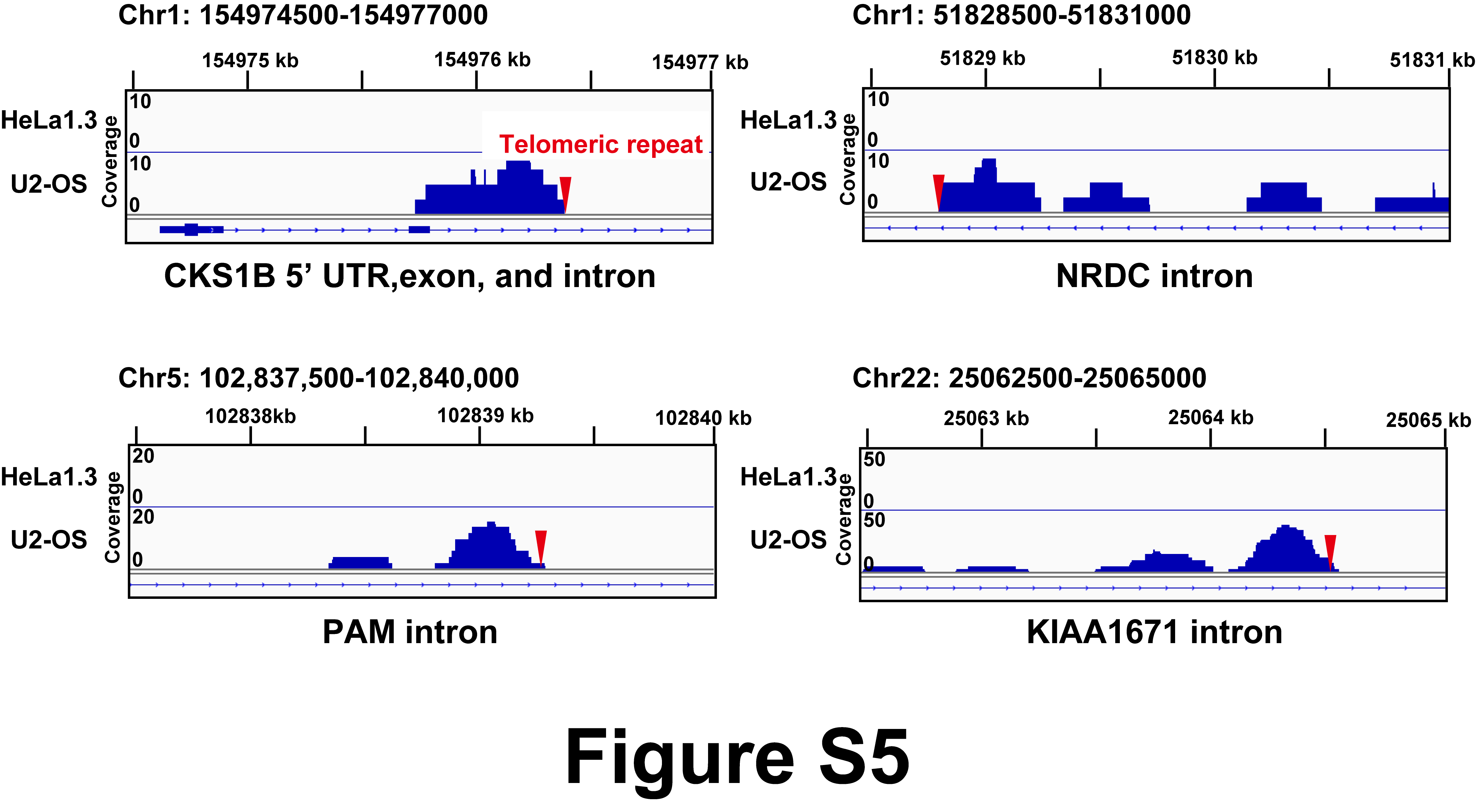

Supplement: Supplementary file 5 — Additional file 5: Figure S5. DNA sequence analysis of TH59-DB binding sites. Genomic regions coding the introns of CKS1B, NRDC, PAM, and KIAA1671 were specifically enriched in the TH59-DB pull-down fraction from ALT U2-OS cells. These intron regions were enriched in the TH59-DB fraction from U2-OS, but not in HeLa1.3 cells. The positions of the inserted telomeric repeats are indicated by red arrowheads. [file 13072_2021_421_MOESM5_ESM.tif]

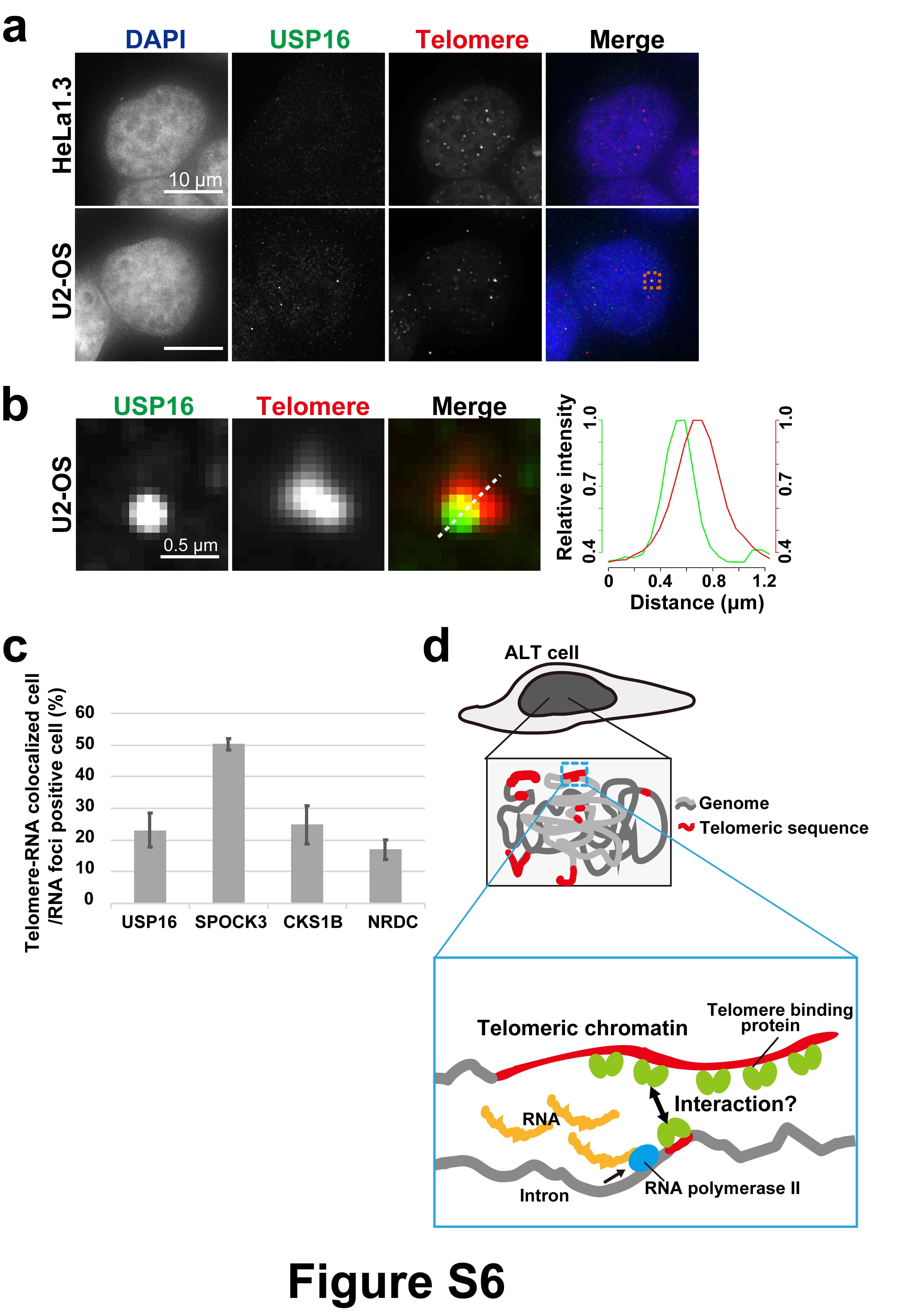

Supplement: Supplementary file 6 — Additional file 6: Figure S6. RNA-FISH for intronic ncRNAs identified by PI-PRICh in ALT cells and a model for chromatin tethering to telomeres with ncRNAs. a RNA-FISH for the USP16 intron and the simultaneous telomere labeling with the fluorescent TH59 probe. First column, DAPI signal; second column, ncRNA signal of TERRA; third column, fluorescent TH59 signal (telomere); fourth column, merged image of DAPI (blue), ncRNA (green) and telomere (red). b Enlarged images of the boxed region in a. Line plot of the USP16-intronic RNA signal and the telomeric signal on the white dotted line in the left merged image. c Bar graph of percentages of cells in which USP16, SPOCK3, CKS1B and NRDC intronic RNA signals were co-localized with telomere signal. Three independent experiments were performed and the numbers of ncRNA signals measured for U2-OS cells were from 100 cells for each experiment. Error bars show standard deviation. d A model of how the genome region with inserted telomeric repeats tethers to telomere in ALT cells. [file 13072_2021_421_MOESM6_ESM.tif]
